# Crystal structure of *rac*-2,3-diphenyl-2,3,5,6-tetrahydro-4*H*-1,3-thiazin-4-one 1-oxide

**DOI:** 10.1107/S2056989016015395

**Published:** 2016-10-07

**Authors:** Hemant P. Yennawar, Ziwei Yang, Lee J. Silverberg

**Affiliations:** a Department of Biochemistry and Molecular Biology, Pennsylvania State University, University Park, PA 16802, USA; b Pennsylvania State University, Schuylkill Campus, 200 University Drive, Schuylkill Haven, PA 17972, USA

**Keywords:** crystal structure, thia­zine compound, envelope pucker

## Abstract

In the title compound, C_16_H_15_NO_2_S [alternative name: *rac*-2,3-diphenyl-1,3-thia­zinan-4-one 1-oxide], the thia­zine ring exhibits an envelope conformation, with the S atom forming the flap. The sulfoxide O atom is in a pseudo-axial position on the thia­zine ring and is *trans* to the phenyl group on C-2. The phenyl rings form a dihedral angle of 89.47 (19)°. In this racemate crystal, homochiral mol­ecules form slabs parallel to (010) of thickness *b*/2 which then stack with alternating chirality in the *b*-axis direction. The stacking is aided by edge-to-face inter­actions between the phenyl rings of racemic mol­ecules. Within each of the single-enanti­omer slabs, the mol­ecules are held by C—H⋯O-type inter­actions, with an H⋯O distance of 2.30 Å, forming infinite chains along the *c*-axis direction, as well by the edge-to-face inter­actions between phenyl rings of neighboring mol­ecules in the *a*-axis direction.

## Abstract 2

In the title compound, the thia­zine ring exhibits an envelope conformation, with the S atom forming the flap of the envelope. In this racemate crystal, homochiral mol­ecules form slabs parallel to (010) of thickness *b*/2 which then stack with alternating chirality in the *b-axis* direction. The stacking is aided by edge-to-face inter­actions between the phenyl rings of racemic mol­ecules.

## Chemical context   

1,3-Thia­zin-4-ones are a group of six-membered heterocycles with a wide range of biological activity (Ryabukhin *et al.*, 1996 ▸). Surrey’s research (Surrey *et al.*, 1958 ▸; Surrey, 1963*a* ▸) resulted in the discovery of two drugs, the anti-anxiety and muscle relaxant chlormezanone [2-(4-chloro­phen­yl)-3-methyl-2,3,5,6-tetra­hydro-4*H*-1,3-thia­zin-4-one 1,1-dioxide; Merck Index, 2006 ▸; Tanaka & Hirayama, 2005 ▸] and muscle relaxant dichlormezanone [2-(3,4-di­chloro­phen­yl)-3-methyl-2,3,5,6-tetra­hydro-4*H*-1,3-thia­zin-4-one 1,1-dioxide; Dictionary of Drugs, 1990 ▸]. These sulfones showed greater activity than the sulfides from which they were synthesized (Surrey *et al.*, 1958 ▸). Surrey also prepared a variety of other sulfoxides and sulfones of 3-alkyl-2-aryl-2,3,5,6-tetra­hydro-4*H*-1,3-thia­zin-4-ones (Surrey, 1963*a* ▸,*b* ▸). Surrey did not successfully synthesize any 2-aryl-3-aryl-2,3,5,6-tetra­hydro-4*H*-1,3-thia­zin-4-ones (Silverberg *et al.*, 2015 ▸), and to the best of our knowledge nobody has reported any oxides of this type of compound. We previously reported the crystal structure of 2,3-diphenyl-2,3,5,6-tetra­hydro-4*H*-1,3-thia­zin-4-one (Yennawar & Silverberg, 2014 ▸). Herein, we report the crystal structure of that compound’s sulfoxide, prepared using the method we have previously reported for oxidation of the five-membered 1,3-thia­zolidin-4-ones (Cannon *et al.*, 2015 ▸).

## Structural commentary   

**Figure 1:**
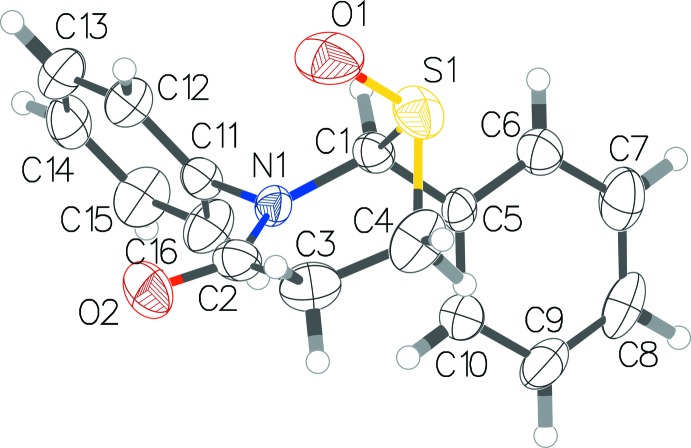
The mol­ecular conformation and atom-numbering scheme for the title compound, with non-H atoms shown as 50% probability displacement ellipsoids.

The crystal structure of this racemic compound shows a thia­zine ring in an envelope pucker with puckering amplitude of 0.718 (3) Å (Fig. 1 ▸). The oxygen on sulfur is pseudo-axial on the thia­zine ring. The two phenyl rings, on two adjacent atoms of the thia­zine ring, are perpendicular to each other with an angle of 89.47 (19)° between their planes. The oxygen on sulfur and the phenyl ring on C2 are *trans* to each other.
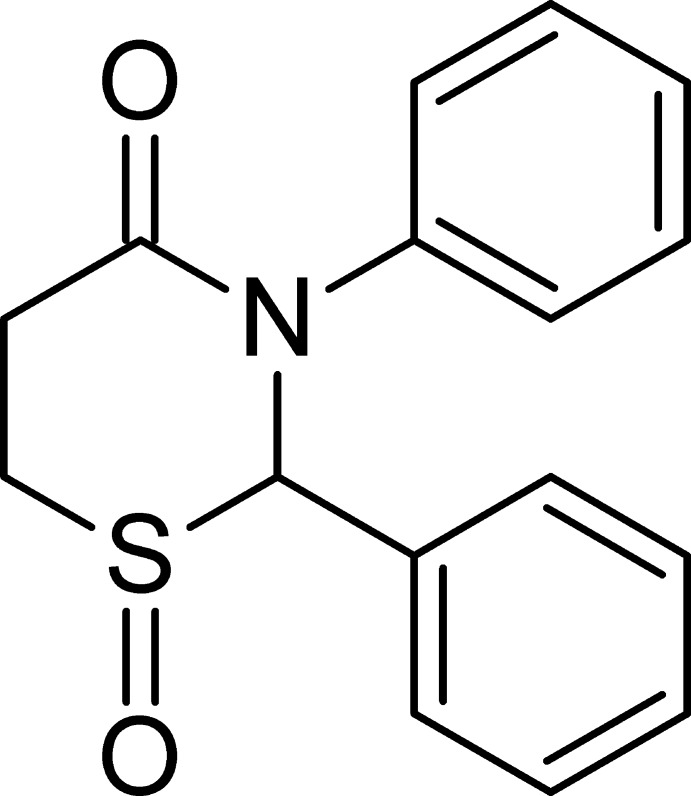


## Supra­molecular features   

**Figure 2:**
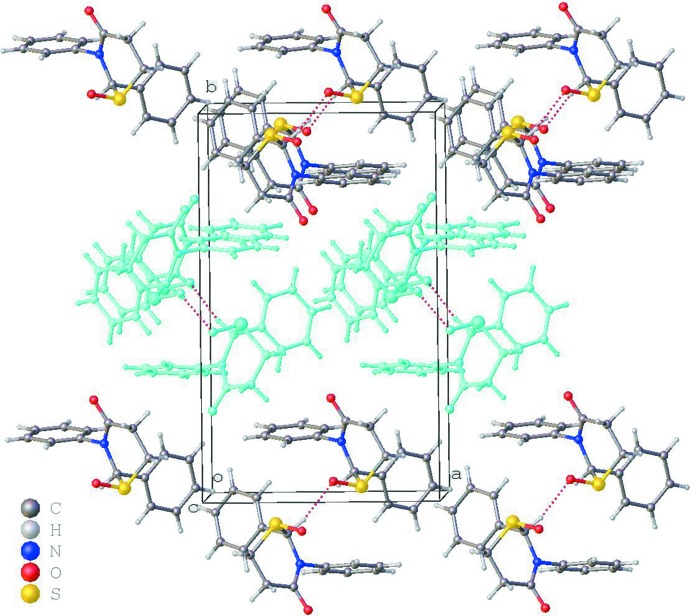
Packing viewed down the *c* axis. Alternating slabs of enanti­omers along the *b*-axis direction are differentiated by the color scheme.

The crystal consists of a racemic mixture of the title compound. The two phenyl groups and one of the two oxygen atoms participate in inter­molecular inter­actions (Table 1 ▸). The mol­ecules of single chirality form slabs in the *ac* plane aided by π–π edge-to-face inter­actions, with inter-centroid distance of 5.195 (3) Å, in the *a*-axis direction and with C—H⋯O hydrogen-bonds (Table 1 ▸) in the *c*-axis direction (Fig. 2 ▸). Along the *b*-axis direction, these slabs stack with alternating chirality, stabilized once again by π–π edge-to-face inter­actions with inter-centroid distances of 5.021 (3) Å.

## Database survey   

Crystal structures of a number of 1,3-thia­zolidin-4-one 1-oxides have been reported (Wang *et al.*, 2010 ▸; Johnson *et al.*, 1983 ▸; Chen *et al.*, 2011 ▸; Colombo *et al.*, 2008 ▸; Yennawar *et al.*, 2015 ▸); in each case the oxygen on sulfur and the group on C-2 had a *trans* relationship, as does the structure reported here. The structure of chlormezanone [2-(4-chloro­phen­yl)-3-methyl-2,3,5,6-tetra­hydro-4*H*-1,3-thia­zin-4-one 1,1-dioxide] has also been disclosed (Tanaka & Horayama, 2005 ▸). To the best of our knowledge, there have been no published crystal structures of a 2,3,5,6-tetra­hydro-4*H*-1,3-thia­zin-4-one 1-oxide.

## Synthesis and crystallization   

A 5 mL round-bottom flask was charged with 50.5 mg of 2,3-diphenyl-2,3,5,6-tetra­hydro-4*H*-1,3-thia­zin-4-one and 1.5 mL of methanol and stirred. A solution of 85.6 mg Oxone^®^ and 0.74 mL distilled water was added dropwise and the mixture was stirred until the reaction was complete as determined by TLC. The solids were dissolved by addition of 7.4 mL distilled water. The solution was extracted with 7.4 mL di­chloro­methane. The organic layer was washed with distilled water and then with sat. sodium chloride. The solution was dried over Na_2_SO_4_ and concentrated under vacuum to a crude solid. This was chromatographed on flash silica gel, eluting with 70% ethyl acetate/hexa­nes, 100% ethyl acetate, and 100% acetone, giving 37.5 mg product (70% yield), m.p.: 396–400 K, *R_f_* = 0.23 (EtOAc). Crystals for X-ray crystallography were grown by slow evaporation from ethanol solution.

## Refinement   

Crystal data, data collection and structure refinement details are summarized in Table 2 ▸. The hydrogen atoms were placed geometrically and allowed to ride on the carbon atoms during refinement, with C—H distances of 0.98 Å (methine), 0.96 Å (meth­yl) and 0.93 Å (aromatic) and with *U*_iso_(H) = 1.2*U*_eq_(aromatic and methine C) or 1.5*U*_eq_(meth­yl).

## Supplementary Material

Crystal structure: contains datablock(s) I. DOI: 10.1107/S2056989016015395/gk2665sup1.cif

Structure factors: contains datablock(s) I. DOI: 10.1107/S2056989016015395/gk2665Isup2.hkl

Supporting information file. DOI: 10.1107/S2056989016015395/gk2665Isup3.mol

Supporting information file. DOI: 10.1107/S2056989016015395/gk2665Isup4.cml

CCDC reference: 1507647

Additional supporting information: crystallographic information; 3D view; checkCIF report

## Figures and Tables

**Table 1:** Hydrogen-bond geometry (Å, °)

*D*—H⋯*A*	*D*—H	H⋯*A*	*D*⋯*A*	*D*—H⋯*A*
C1—H1⋯O1^i^	0.98	2.30	3.261 (5)	167

Symmetry code: (i) 
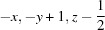
.

**Table 2:** Experimental details

Crystal data	
Chemical formula	C_16_H_15_NO_2_S
*M*_r_	285.35
Crystal system, space group	Orthorhombic, *P* *n* *a*2_1_
Temperature (K)	298
*a*, *b*, *c* (Å)	10.547 (4), 17.317 (6), 7.592 (3)
*V* (Å^3^)	1386.5 (8)
*Z*	4
Radiation type	Mo *K*α
μ (mm^−1^)	0.23
Crystal size (mm)	0.23 × 0.18 × 0.16

Data collection	
Diffractometer	Bruker SMART APEX CCD area detector
Absorption correction	Multi-scan (*SADABS*; Bruker, 2001 ▸)
*T*_min_, *T*_max_	0.636, 0.964
No. of measured, independent and observed [*I* > 2σ(*I*)] reflections	11124, 3428, 3182
*R*_int_	0.032
(sin θ/λ)_max_ (Å^−1^)	0.668

Refinement	
*R*[*F* ^2^ > 2σ(*F* ^2^)], *wR*(*F* ^2^), *S*	0.075, 0.161, 1.26
No. of reflections	3428
No. of parameters	181
No. of restraints	1
H-atom treatment	H-atom parameters constrained
Δρ_max_, Δρ_min_ (e Å^−3^)	0.38, −0.27
Absolute structure	Flack (1983 ▸), 4716 Friedel pairs
Absolute structure parameter	0.12 (14)

Computer programs: *SMART* and *SAINT* (Bruker, 2001 ▸), *SHELXS97* (Sheldrick, 2008 ▸), *SHELXL2014* (Sheldrick, 2015 ▸) and *OLEX2* (Dolomanov *et al.*, 2009 ▸).

## supplementary crystallographic information

### Crystal data

**Table:**

C_16_H_15_NO_2_S	*D*_x_ = 1.367 Mg m^−^^3^
*M**_r_* = 285.35	Mo *K*α radiation, λ = 0.71073 Å
Orthorhombic, *P**n**a*2_1_	Cell parameters from 3661 reflections
*a* = 10.547 (4) Å	θ = 2.3–26.4°
*b* = 17.317 (6) Å	µ = 0.23 mm^−^^1^
*c* = 7.592 (3) Å	*T* = 298 K
*V* = 1386.5 (8) Å^3^	Block, colorless
*Z* = 4	0.23 × 0.18 × 0.16 mm
*F*(000) = 600	

### Data collection

**Table:**

Bruker SMART APEX CCD area detector diffractometer	3428 independent reflections
Radiation source: fine-focus sealed tube	3182 reflections with *I* > 2σ(*I*)
Parallel,graphite monochromator	*R*_int_ = 0.032
φ and ω scans	θ_max_ = 28.3°, θ_min_ = 2.3°
Absorption correction: multi-scan (SADABS; Bruker, 2001)	*h* = −13→13
*T*_min_ = 0.636, *T*_max_ = 0.964	*k* = −20→23
11124 measured reflections	*l* = −10→10

### Refinement

**Table:**

Refinement on *F*^2^	Secondary atom site location: difference Fourier map
Least-squares matrix: full	Hydrogen site location: inferred from neighbouring sites
*R*[*F*^2^ > 2σ(*F*^2^)] = 0.075	H-atom parameters constrained
*wR*(*F*^2^) = 0.161	*w* = 1/[σ^2^(*F*_o_^2^) + (0.0582*P*)^2^ + 0.8049*P*] where *P* = (*F*_o_^2^ + 2*F*_c_^2^)/3
*S* = 1.26	(Δ/σ)_max_ < 0.001
3428 reflections	Δρ_max_ = 0.38 e Å^−^^3^
181 parameters	Δρ_min_ = −0.27 e Å^−^^3^
1 restraint	Absolute structure: Flack (1983), 4716 Friedel pairs
Primary atom site location: structure-invariant direct methods	Absolute structure parameter: 0.12 (14)

### Special details

**Table:**

Experimental. 1. SADABS was used for absorption correction. R(int) was 0.0424 before and 0.0268 after correction. The Ratio of minimum to maximum transmission is 0.6364. The λ/2 correction factor is 0.0015.2. The data collection nominally covered a full sphere of reciprocal space by a combination of 4 sets of ω scans each set at different φ and/or 2θ angles and each scan (5 s exposure) covering -0.300° degrees in ω. The crystal to detector distance was 5.82 cm.
Geometry. All esds (except the esd in the dihedral angle between two l.s. planes) are estimated using the full covariance matrix. The cell esds are taken into account individually in the estimation of esds in distances, angles and torsion angles; correlations between esds in cell parameters are only used when they are defined by crystal symmetry. An approximate (isotropic) treatment of cell esds is used for estimating esds involving l.s. planes.
Refinement. Refinement of F^2^ against ALL reflections. The weighted R-factor wR and goodness of fit S are based on F^2^, conventional R-factors R are based on F, with F set to zero for negative F^2^. The threshold expression of F^2^ > 2sigma(F^2^) is used only for calculating R-factors(gt) etc. and is not relevant to the choice of reflections for refinement. R-factors based on F^2^ are statistically about twice as large as those based on F, and R- factors based on ALL data will be even larger.

### Fractional atomic coordinates and isotropic or equivalent isotropic displacement parameters (Å^2^)

**Table:**

	*x*	*y*	*z*	*U*_iso_*/*U*_eq_	
S1	0.17133 (10)	0.45935 (5)	−1.97816 (14)	0.0469 (3)	
O1	0.0575 (3)	0.44303 (18)	−1.8700 (4)	0.0659 (9)	
O2	0.0431 (3)	0.23452 (16)	−2.0769 (4)	0.0542 (8)	
N1	0.0796 (2)	0.34956 (15)	−2.2011 (4)	0.0281 (6)	
C1	0.1326 (3)	0.42779 (19)	−2.2029 (4)	0.0304 (7)	
H1	0.0646	0.4619	−2.2442	0.036*	
C2	0.1038 (4)	0.2938 (2)	−2.0808 (5)	0.0375 (8)	
C3	0.2109 (4)	0.3050 (2)	−1.9494 (5)	0.0483 (10)	
H3A	0.1771	0.2936	−1.8334	0.058*	
H3B	0.2745	0.2660	−1.9744	0.058*	
C4	0.2783 (4)	0.3813 (3)	−1.9375 (5)	0.0521 (11)	
H4A	0.3465	0.3828	−2.0231	0.063*	
H4B	0.3151	0.3870	−1.8211	0.063*	
C5	0.2437 (3)	0.44151 (17)	−2.3232 (4)	0.0278 (6)	
C6	0.2671 (4)	0.5164 (2)	−2.3791 (5)	0.0392 (8)	
H6	0.2126	0.5559	−2.3446	0.047*	
C7	0.3688 (4)	0.5333 (3)	−2.4840 (8)	0.0534 (10)	
H7	0.3842	0.5839	−2.5186	0.064*	
C8	0.4485 (4)	0.4748 (3)	−2.5384 (6)	0.0517 (11)	
H8	0.5175	0.4860	−2.6103	0.062*	
C9	0.4261 (3)	0.4006 (2)	−2.4869 (6)	0.0472 (9)	
H9	0.4799	0.3613	−2.5247	0.057*	
C10	0.3245 (3)	0.3830 (2)	−2.3791 (5)	0.0391 (8)	
H10	0.3102	0.3323	−2.3441	0.047*	
C11	−0.0254 (3)	0.33887 (18)	−2.3192 (4)	0.0297 (7)	
C12	−0.1478 (4)	0.3459 (2)	−2.2599 (5)	0.0408 (8)	
H12	−0.1627	0.3562	−2.1415	0.049*	
C13	−0.2485 (4)	0.3378 (2)	−2.3733 (6)	0.0477 (10)	
H13	−0.3313	0.3424	−2.3323	0.057*	
C14	−0.2254 (4)	0.3231 (2)	−2.5468 (5)	0.0439 (9)	
H14	−0.2934	0.3171	−2.6236	0.053*	
C15	−0.1043 (4)	0.3169 (3)	−2.6103 (5)	0.0492 (10)	
H15	−0.0901	0.3077	−2.7294	0.059*	
C16	−0.0032 (4)	0.3244 (2)	−2.4953 (5)	0.0454 (9)	
H16	0.0794	0.3197	−2.5366	0.055*	

### Atomic displacement parameters (Å^2^)

**Table:**

	*U*^11^	*U*^22^	*U*^33^	*U*^12^	*U*^13^	*U*^23^
S1	0.0600 (6)	0.0427 (5)	0.0380 (4)	−0.0080 (4)	0.0072 (5)	−0.0128 (4)
O1	0.083 (2)	0.0605 (18)	0.0548 (19)	0.0131 (17)	0.0259 (18)	−0.0026 (16)
O2	0.0614 (18)	0.0366 (15)	0.0648 (19)	−0.0055 (13)	0.0023 (15)	0.0188 (13)
N1	0.0242 (13)	0.0277 (13)	0.0325 (13)	−0.0015 (10)	0.0013 (12)	0.0054 (11)
C1	0.0326 (16)	0.0262 (15)	0.0323 (16)	0.0012 (13)	−0.0013 (14)	0.0026 (13)
C2	0.041 (2)	0.0306 (17)	0.0410 (19)	0.0064 (14)	0.0042 (16)	0.0056 (15)
C3	0.049 (2)	0.055 (2)	0.041 (2)	0.0142 (18)	−0.0079 (18)	0.0038 (18)
C4	0.042 (2)	0.080 (3)	0.034 (2)	−0.003 (2)	−0.0060 (16)	0.0044 (18)
C5	0.0233 (14)	0.0287 (14)	0.0316 (16)	−0.0045 (12)	−0.0070 (12)	0.0007 (13)
C6	0.0376 (19)	0.0357 (17)	0.0441 (19)	−0.0002 (15)	−0.0024 (16)	0.0036 (16)
C7	0.046 (2)	0.057 (2)	0.057 (2)	−0.0126 (18)	0.003 (3)	0.018 (2)
C8	0.0308 (19)	0.081 (3)	0.043 (2)	−0.017 (2)	0.0064 (16)	0.006 (2)
C9	0.0275 (16)	0.064 (2)	0.051 (2)	−0.0013 (16)	0.0053 (19)	−0.012 (2)
C10	0.0344 (19)	0.0370 (17)	0.046 (2)	−0.0008 (14)	−0.0017 (16)	−0.0019 (17)
C11	0.0333 (17)	0.0266 (14)	0.0293 (15)	0.0000 (12)	−0.0056 (14)	−0.0002 (13)
C12	0.0364 (19)	0.047 (2)	0.0388 (19)	−0.0003 (16)	0.0021 (15)	0.0013 (17)
C13	0.0287 (18)	0.055 (2)	0.059 (2)	−0.0006 (17)	0.0004 (18)	−0.002 (2)
C14	0.042 (2)	0.040 (2)	0.050 (2)	−0.0065 (16)	−0.0185 (17)	0.0007 (17)
C15	0.048 (2)	0.073 (3)	0.0261 (17)	−0.004 (2)	−0.0027 (17)	−0.0071 (18)
C16	0.0369 (18)	0.062 (2)	0.0374 (19)	−0.0022 (16)	0.0089 (17)	−0.0001 (19)

### Geometric parameters (Å, º)

**Table:**

S1—O1	1.482 (3)	C5—C10	1.390 (5)
S1—C1	1.838 (4)	C6—C7	1.368 (6)
S1—C4	1.787 (5)	C7—C8	1.379 (6)
O2—C2	1.210 (5)	C8—C9	1.364 (6)
N1—C1	1.466 (4)	C9—C10	1.383 (5)
N1—C2	1.354 (4)	C11—C12	1.372 (5)
N1—C11	1.436 (4)	C11—C16	1.380 (5)
C1—C5	1.505 (5)	C12—C13	1.375 (6)
C2—C3	1.519 (5)	C13—C14	1.363 (6)
C3—C4	1.503 (6)	C14—C15	1.370 (6)
C5—C6	1.387 (5)	C15—C16	1.384 (6)
			
O1—S1—C1	106.12 (18)	C6—C5—C10	118.7 (3)
O1—S1—C4	105.8 (2)	C10—C5—C1	123.2 (3)
C4—S1—C1	94.34 (17)	C7—C6—C5	121.1 (4)
C2—N1—C1	126.4 (3)	C6—C7—C8	119.7 (4)
C2—N1—C11	118.4 (3)	C9—C8—C7	120.1 (4)
C11—N1—C1	114.0 (2)	C8—C9—C10	120.7 (4)
N1—C1—S1	110.6 (2)	C9—C10—C5	119.7 (3)
N1—C1—C5	116.7 (3)	C12—C11—N1	120.6 (3)
C5—C1—S1	110.1 (2)	C12—C11—C16	119.5 (3)
O2—C2—N1	121.4 (3)	C16—C11—N1	119.8 (3)
O2—C2—C3	119.0 (3)	C11—C12—C13	120.8 (4)
N1—C2—C3	119.5 (3)	C14—C13—C12	119.1 (4)
C4—C3—C2	120.2 (3)	C13—C14—C15	121.4 (4)
C3—C4—S1	110.9 (3)	C14—C15—C16	119.3 (4)
C6—C5—C1	118.1 (3)	C11—C16—C15	119.9 (3)
			
S1—C1—C5—C6	−76.3 (3)	C2—N1—C11—C16	−109.6 (4)
S1—C1—C5—C10	102.9 (3)	C2—C3—C4—S1	34.5 (4)
O1—S1—C1—N1	−49.2 (3)	C4—S1—C1—N1	58.6 (3)
O1—S1—C1—C5	−179.5 (2)	C4—S1—C1—C5	−71.7 (3)
O1—S1—C4—C3	46.9 (3)	C5—C6—C7—C8	1.3 (7)
O2—C2—C3—C4	−173.1 (4)	C6—C5—C10—C9	0.6 (5)
N1—C1—C5—C6	156.6 (3)	C6—C7—C8—C9	−0.3 (7)
N1—C1—C5—C10	−24.1 (4)	C7—C8—C9—C10	−0.5 (7)
N1—C2—C3—C4	8.0 (5)	C8—C9—C10—C5	0.3 (6)
N1—C11—C12—C13	178.2 (3)	C10—C5—C6—C7	−1.4 (6)
N1—C11—C16—C15	−177.7 (3)	C11—N1—C1—S1	139.1 (2)
C1—S1—C4—C3	−61.2 (3)	C11—N1—C1—C5	−94.1 (3)
C1—N1—C2—O2	170.2 (3)	C11—N1—C2—O2	3.5 (5)
C1—N1—C2—C3	−11.0 (5)	C11—N1—C2—C3	−177.6 (3)
C1—N1—C11—C12	−95.3 (4)	C11—C12—C13—C14	−0.3 (6)
C1—N1—C11—C16	82.2 (4)	C12—C11—C16—C15	−0.1 (6)
C1—C5—C6—C7	177.9 (4)	C12—C13—C14—C15	−0.6 (6)
C1—C5—C10—C9	−178.7 (3)	C13—C14—C15—C16	1.1 (7)
C2—N1—C1—S1	−28.1 (4)	C14—C15—C16—C11	−0.8 (6)
C2—N1—C1—C5	98.7 (4)	C16—C11—C12—C13	0.7 (6)
C2—N1—C11—C12	72.9 (4)		

### Hydrogen-bond geometry (Å, º)

**Table:**

*D*—H···*A*	*D*—H	H···*A*	*D*···*A*	*D*—H···*A*
C1—H1···O1^i^	0.98	2.30	3.261 (5)	167

Symmetry code: (i) −*x*, −*y*+1, *z*−1/2.
